# Advances in Microbial and Nucleic Acids Biotechnology

**DOI:** 10.1155/2018/3102374

**Published:** 2018-06-24

**Authors:** Gamal Enan, Mohamed E. Osman, Mahmoud E. F. Abdel-Haliem, Salah E. Abdel-Ghany

**Affiliations:** ^1^Department of Botany and Microbiology, Faculty of Science, Zagazig University, Zagazig 44519, Egypt; ^2^Department of Botany and Microbiology, Helwan University, Cairo, Egypt; ^3^Department of Biology, Colorado State University, Fort Collins, CO 80523, USA

Biotechnology in its broad meaning is the use of organisms or their products for various purposes such as fast diagnosis of infectious diseases, inhibition of the antibiotic resistant bacteria, production of pharmaceuticals, biomaterials, and even energetic materials. Recent challenges in Biotechnology offer state-of-the art studies covering a wide spectrum of biotechnological applications in different organisms for different purposes that serve agriculture, environment, health, and industry. Genomes of both eukaryotes and prokaryotes are recently engineered for advances in food safety and development in all fields.

The call for submission of manuscripts allowed researchers in Biotechnology fields to submit their original and novel findings to this special issue. Nineteen manuscripts were submitted. According to precise peer review processes as well as the policies and standards applied in the journal, only seven of them were accepted and they are in the context of this special issue. The full papers in this issue are categorized in four different subjects based on the scope of the Biotechnology applications.

Three papers in this special issue discussed results in the immunotherapeutic field. The paper titled “Mannose-Binding Lectin: A Potential Therapeutic Candidate against* Candida* Infection” showed the potential therapeutic capability of mannose binding lectin (MBL) against candidiasis as this recombinant MBL induced agglutination of both* Candida albicans* and* C. glabrata*. In their extended studies in the paper titled “Mannose-Binding Lectin Gene Polymorphism and Its Association with Susceptibility to Recurrent Vulvovaginal Candidiasis,” the authors studied the MBL gene polymorphism and its association with recurrent vulvovaginal candidiasis (RVVC) and found a close correlation between innate immunity gene mutation and polymorphism in MBL gene and the existence of RVVC. They concluded that MBL genotypic analysis can be used as a surrogate for MBL serum level in order to identify MBL-deficient women for alterative therapeutic options. In the same category, the paper titled “Microcrystalline Cellulose for Delivery of Recombinant Protein-Based Antigen against Erysipelas in Mice” is very interesting as it developed microcrystalline cellulose for delivery of recombinant protein-based antigen against erysipelas in mice. This recombinant surface protein (SPA) from the Gram positive pathogen* Erysipelothrix rhusiopathiae* was fused to cellulose binding domain from* Trichoderma harzianum* (CBD). This CBD-SPA fusion cassette was expressed in* E. coli* successfully.

Molecular cloning and gene expression category include 2 papers. In the paper titled “*Lra*I from* Lactococcus raffinolactis* BGTRK10-1, an Isoschizomer of* Eco*RI, Exhibits Ion Concentration-Dependent Specific Star Activity,” the results obtained found that* Lactococcus raffinolactis* BG TRK10-1 produces a novel* Lra*I type II restriction endonuclease which is an isoschizomer of* Eco*R1, and the gene encoding its production was cloned and expressed successfully in the* E. coli* bacterium. In the other paper of this category titled “Cloning and Expression of the Organophosphate Pesticide-Degrading* α-β* Hydrolase Gene in Plasmid pMK-07 to Confer Cross-Resistance to Antibiotics,” the authors were able to clone and express the *α*-*β*-hydrolase gene in some strains of bacteria belonging to the genus* Bacillus*; this is a promising result since* α-β* hydrolase produced by bacteria degrades the organophosphate pesticide pollutants in soil.

Food Biotechnology area of research includes one paper only titled “Genetic Analysis with Random Amplified Polymorphic DNA of the Multiple Enterocin-Producing* Enterococcus lactis* 4CP3 Strain and Its Efficient Role on the Growth of* Listeria monocytogenes* in Raw Beef Meat”; the results obtained showed that the* Enterococcus lactis* 4CP3 strain could be used as meat protective against listerias growth at refrigeration temperatures. The intraspecific genetic analysis of the 4CP3 strain was assessed by random amplified polymorphic DNA polymerase chain reaction analysis.

Tissue culture Biotechnology includes one paper titled “Manipulation of Plant Growth Regulators on Phytochemical Constituents and DNA Protection Potential of the Medicinal Plant* Arnebia benthamii*” which showed a novel protocol for the* in vitro* regeneration of the medicinal plant* Arnebia benthamii* by tissue culture techniques. It was found that the regenerated plants possessed high content of volatile/nonvolatile compounds that showed DNA protection potential against oxidants.

*Gamal Enan* *Mohamed E. Osman* *Mahmoud E. F. Abdel-Haliem* *Salah E. Abdel-Ghany*

